# Unraveling the periprandial changes in brain serotonergic activity and its correlation with food intake-related neuropeptides in rainbow trout *Oncorhynchus mykiss*

**DOI:** 10.3389/fendo.2023.1241019

**Published:** 2023-08-24

**Authors:** Mauro Chivite, Rosa M. Ceinos, José M. Cerdá-Reverter, Jose L. Soengas, Manuel Aldegunde, Marcos A. López-Patiño, Jesús M. Míguez

**Affiliations:** ^1^ Centro de Investigación Mariña, Laboratorio de Fisioloxía Animal, Departamento de Bioloxía Funcional e Ciencias da Saúde, Facultade de Bioloxía, Universidade de Vigo, Vigo, Spain; ^2^ Departamento de Fisiología de Peces y Biotecnología, Instituto de Acuicultura Torre de la Sal, Instituto de Acuicultura Torre de la Sal - Consejo Superior de Investigaciones Científicas (IATS-CSIC), Castellón, Spain; ^3^ Departamento de Fisiología, Facultad de Biología, Universidad de Santiago de Compostela, Santiago de Compostela, Spain

**Keywords:** feeding regulation, serotonin, neuropeptides, hypothalamus, brain, trout, teleost

## Abstract

This study explored changes in brain serotonin content and activity together with hypothalamic neuropeptide mRNA abundance around feeding time in rainbow trout, as well as the effect of one-day fasting. Groups of trout fed at two (ZT2) and six (ZT6) hours after lights on were sampled from 90 minutes before to 240 minutes after feeding, while additional groups of non-fed trout were also included in the study. Changes in brain amine and metabolite contents were measured in hindbrain, diencephalon and telencephalon, while in the diencephalon the mRNA abundance of tryptophan hydroxylase (*tph1*, *tph2*), serotonin receptors *(5htr1a*, *5htr1b* and *5htr2c*) and several neuropeptides (*npy*, *agrp1*, *cartpt*, *pomca1*, *crfb*) involved in the control of food intake were also assessed. The results showed changes in the hypothalamic neuropeptides that were consistent with the expected role for each in the regulation of food intake in rainbow trout. Serotonergic activity increased rapidly at the time of food intake in the diencephalon and hindbrain and remained high for much of the postprandial period. This increase in serotonin abundance was concomitant with elevated levels of *pomca1* mRNA in the diencephalon, suggesting that serotonin might act on brain neuropeptides to promote a satiety profile. Furthermore, serotonin synthesis and neuronal activity appear to increase already before the time of feeding, suggesting additional functions for this amine before and during food intake. Exploration of serotonin receptors in the diencephalon revealed only small changes for gene expression of *5htr1b* and *5htr2c* receptors during the postprandial phase. Therefore, the results suggest that serotonin may play a relevant role in the regulation of feeding behavior in rainbow trout during periprandial time, but a better understanding of its interaction with brain centers involved in receiving and processing food-related signals is still needed.

## 1 Introduction

Feeding behavior involves the coordination of multiple complex neural circuits that allow the animal to obtain food at the appropriate times and regulate the amount it eats. In vertebrates, sensory information related to feeding status, whether from olfactory and/or gustatory receptors, gastrointestinal digestive processes, circulating nutrient levels or the individual’s own behavior, are integrated at the central nervous system and activates stimulatory/inhibitory outputs that control food intake (1–3). Two populations of neuropeptidergic neurons of the hypothalamus are involved in the control of food intake in mammals, the first expressing the orexigenic neuropeptide Y (NPY) and agouti-related peptide (AgRP), and the second expressing the anorexigenic proopiomelacortin (POMC) and cocaine- and amphetamine-regulated transcript (CART) (4, 5). In fish, the existence of homologous neuronal populations is still under debate, but orexigenic and anorexigenic effects of neuropeptides have been reported for several fish species (see rev. 6, 7). In addition, circadian signals also modulate feeding behavior by acting through different peripheral and central regulatory pathways in order to accommodate food intake to certain times of the day that are more conducive from the point of view of digestive activity, energy homeostasis and adaptation to the environment (6, 8). Accordingly, fish subjected to a programmed feeding regime periodically increase or decrease the abundance of factors that regulate appetite and satiety, respectively, with these changes coinciding mostly with the preprandial and postprandial phases (3).

In addition to neuropeptides, several brain neurotransmitters are involved in feeding regulation, including the monoamines (9, 10). Serotonin (5-hydroxytryptamine, 5HT) is widely distributed in central nervous system and is one of the most important neurotransmitters affecting the regulation of food intake (11). 5HT synthesis takes place from the amino acid L-tryptophan, which is first hydroxylated by tryptophan hydroxylase (TPH, EC 1.14.16.4) to form 5-hydroxytryptophan (5HTP), and then decarboxylated by L-aromatic acid decarboxylase (AADC, 4.1.1.28) to give serotonin. Due to the rapid activity of AADC, the levels of 5HTP are usually low, and TPH is thought to act as the rate-limiting enzyme of 5HT biosynthesis (12). Once the serotonergic neuron is activated, the amine stored in vesicles in neuronal terminals is released exocytotically to the synaptic cleft, where it binds to specific 5HT receptors (5HTR), either auto or heteroreceptors (13). Subsequently, sodium-dependent serotonin transporters (SERT or slc6a4) mediate the reuptake of 5HT to neuronal endings, where it is metabolized to 5-hydroxyindoleacetic acid (5HIAA) by the sequential action of monoamine oxidase and aldehyde dehydrogenase (14). 5HT synthesis, reuptake and degradation are well-conserved processes through phylogeny, so that 5HT is one of the most widespread signaling molecules in vertebrates, including fish (15).

Evidence in mammals suggests that the central 5HTergic system acts as a suppressor of food anticipatory activity (FAA) and as a potent satiety signal (16). Multiple studies have focused on 5HTR subtypes involved in feeding regulation, pointing to a priority role of the postsynaptic 5HT_2C_ and 5HT_1B_ receptors and probably the presynaptic 5HT_1A_ receptor (17–19). 5HT_2C_ receptor is widely expressed in the CNS and specifically in brain areas related to energy metabolism, including the ventral tegmental area (VTA), the arcuate nucleus (ARC) and the paraventricular nucleus of the hypothalamus (PVN), among others (20). Of utmost interest is that 5HT_2C_ receptor is co-expressed in hypothalamic (ARC) POMC neurons, which are first-order neurons in the regulation of food intake and energy balance (21). ARC POMC neurons produce melanocyte-stimulating hormone (αMSH), an endogenous agonist of the melanocortin-4 receptor (MC4R) whose activation promotes downstream effects that inhibit food intake (22–24). Additionally, activation of 5HT_1B_ receptors downregulates the firing rate in hypothalamic NPY/AgRP neurons and collaterally increases neuronal release of POMC, which also leads to loss of appetite (10, 21). The presynaptic autoreceptor 5HT_1A_ inhibits serotonergic activity and may led to a down-regulation of the 5HT-mediated appetite inhibition (25).

In fish, monoamine systems are widely spread in brain with major clusters of 5HTergic neurons lying in the diencephalon (26), including the hypothalamic area where the control center for food intake is located (27). Pharmacological studies, i.e., by using 5HT or drugs that modulate serotonergic transmission such as fluoxetine and *d*-fenfluramine administered peripherally or intracerebroventricularly, showed an inhibitory influence of 5HT on food intake (28–30) and weight gain (31). In addition, treatments with serotonergic agonists or antagonists showed a close functional relationship between brain 5HT and changes in the expression of neuropeptides involved in the hypothalamic control of feeding. However, the specific mechanisms are not entirely clear in fish. For instance, the anorectic effect induced by increased 5HT in goldfish brain may be mediated by CRF (28), whereas fluoxetine up-regulated both *crf1* and *npy* gene expression in female goldfish (31). In trout, the decrease in food intake induced by agonistic activation of 5HT_2C_ receptors was followed by an increase in hypothalamic mRNA abundance of *pomca1*, *cart* and *crf* (32–34). Meanwhile, the involvement of 5HT_1B_ receptors in feeding control remains unclear (30).

In teleosts, hypothalamic neuropeptide levels show changes that respond to a number of metabolic, humoral and neural signals that provide information about food intake, nutritional status and circadian timing (35–37). Some of these signals can converge on brain serotonergic neurons at different levels to modulate their activity during feeding time (27, 29, 32). Therefore, 5HT may be a critical element in the interaction with brain neuropeptide circuits, being able to modulate feeding behavior. However, whether there is a temporal relationship between brain 5HTergic activity and the regulatory mechanisms determining daily food intake in teleost remains to be determined. To address this question, the present study addressed in depth the periprandial changes in serotonergic activity and its relationship to feeding regulatory neuropeptides in the brain of fed and non-fed rainbow trout. Since schedule feeding can adjust circadian changes in feeding regulatory systems (38), we assessed changes in 5HT and brain neuropeptides in groups of fish fed on two different daily feeding schedules.

## 2 Materials and methods

### 2.1 Fish

Immature rainbow trout (*Oncorhynchus mykiss*, Walbaum) weighing 92.4 ± 6.4 g, were obtained from a local hatchery (A Estrada, Spain), and transported to the facilities of the Faculty of Biology (University of Vigo). Fish were transferred to 100-L water tanks and began a 20-day acclimation period to laboratory conditions, i.e., 12L:12D light/dark photoperiod, controlled water temperature (13 ± 1°C) and continuously renewed, aerated and dechlorinated fresh water. Trout were fed daily at ZT2 (ZT0 = lights on) with commercial dry pellets (Dibaq-Diproteg SA, Spain; 48% crude protein, 14% carbohydrates, 25% crude fat, and 11.5% ash; 20.2 MJ/kg feed). All the experiments agreed with the European Union Council (2010/63/UE) and Spanish Government (RD 53/2013) Directives for the use of animals in research. The Animal Ethics Committee of the University of Vigo approved all the protocols.

### 2.2 Experimental design and sampling

Following acclimation, two cohorts of fish were randomly distributed into twenty experimental tanks (10 tanks per cohort, 25 individuals per tank), and were fed *ad libitum* for 10 days. Fish belonging to the first cohort received food 2 hours after lights on (zeitgeber time 2, ZT2), whereas those of the second cohort were fed at ZT6. This feeding protocol was applied to discriminate the possible influence of circadian fluctuations of the measured parameters on the changes occurring in relation to the daily feeding time. Each day the fish had visual access to the feeding operator for 5 seconds before the food was delivered. After the training period, trout from six tanks in each cohort were sampled at different times around the respective mealtime (MT): 90 min and 30 min before MT, just at MT (0 min), and 30, 90 and 240 min after MT. To ensure that all the fish of these groups had eaten food, stomach content was analyzed individually at the end of the experiment. The remaining four tanks from each cohort were not fed although a simulated feeding action was performed at mealtime. Trout of the unfed groups were subsequently sacrificed and sampled at 0, 30, 90 and 240 min, relative to the corresponding MT.

Prior to sampling, fish were anesthetized with 0.02% 2-phenoxyethanol (v/v, Merck) pre-diluted in 1 L of water and carefully added to the tank. Only 16 fish from each tank were used, with the remaining 9 fish being transferred to recovery tanks. This surplus of animals *per* experimental tank was established to avoid the incidence of dominance hierarchies, as previously reported (39). Blood samples were collected from the caudal peduncle by puncture with heparinized syringes. Plasma was obtained after blood centrifugation (6,000 g, 10 min at 4° C) and an aliquot of 50 µL was collected and immediately stored at -80° C for cortisol analysis. The remaining plasma volume was deproteinized with 0.6 M perchloric acid followed by centrifugation (14,000 g, 4 min at 4° C) and subsequent neutralization with 1 M potassium bicarbonate, thus frozen (-80° C) for subsequent biochemical determinations. Finally, fish were euthanized by decapitation and brain areas (telencephalon, diencephalon - including the hypothalamus - and hindbrain) were dissected as shown in **Supplementary Figure 1**. Tissues were frozen at -80°C until further analysis. Brain samples from ten fish were analyzed for monoamine content, while those from the remaining six fish were used for q-PCR assays.

### 2.3 Plasma cortisol and metabolites

Plasma cortisol levels were analyzed by a commercial enzyme-linked immunosorbent assay kit (Cayman Chemical Company, Ann Arbor, MI, USA), following the manufacturer’s indications. Plasma glucose levels were measured spectrophotometrically with a commercial kit (Spinreact, Barcelona, Spain).

### 2.4 Brain monoamines

The contents of 5HT and its main oxidative metabolite, the 5HIAA, in brain tissues were assessed by high performance liquid chromatography with electrochemical detection (HPLC-EC) according to Gesto et al. (40), with modifications. Brain tissues were homogenized by ultrasonic disruption in 0.4 mL of HPLC mobile phase and centrifuged (16,000 g, 10 min). Protein content in individual aliquots were estimated by the bicinchoninic acid method, and the remaining volume was filtered (0.2 µm, Millipore) and diluted 1:3 in mobile phase before injected into the HPLC system. This consisted of a Jasco PU-2080 Plus pump and AS-2057 autosampler, a 5-μm reverse phase analytical column (Kinetex C18 100 Å, 150 mm length × 4.6 mm diameter; Phenomenex) maintained at 25° C in a column oven (Jasco CO-4060), and an ESA Coulochem II detector. The detection system included a double analytical cell (M5011) with oxidation potentials set at +40 mV (first electrode) and +340 mV (second electrode). The mobile phase was composed of 63.9 mol/L NaH_2_PO_4_, 0.1 mol/L Na_2_EDTA, 0.80 mol/L sodium 1-octanesulfonate and 14% v/v methanol, and pH adjusted to 2.85 with *o*-phosphoric acid. Mobile phase was filtered, degassed under vacuum and pumped at a flow rate of 1.0 mL/min. The detection limits for 5HT and 5HIAA were 0.5 and 1.5 pg per injection, with a signal-to-noise ratio of 3. ChromNAV version 1.12 software (Jasco Corp.) was used for chromatogram acquisition and data integration.

### 2.5 Quantitative real time PCR (qPCR) assays

Total mRNA was individually extracted from brain samples according to the TRIzol^®^ method (Life Technologies, Grand Island, NY, USA), and treated with DNAse (Promega, Madison, WI, USA). 1 μg total RNA was converted to cDNA with Superscript II reverse transcriptase (Promega) and random hexaprimers (Promega). To identify any genomic contamination in the RNA extract, negative control without reverse transcriptase was run for each sample.

The mRNA abundance was determined by real-time quantitative RT-PCR (q-PCR) using the iCycler iQ™ (BIO-RAD, Hercules, CA, USA). Assays were done in a total PCR volume of 15 μL, containing 2 μL of diluted cDNA (1:8), 7.5 μL of MAXIMA SYBR Green qPCR Mastermix (Life Technologies), 1.0 μL of each primer (0,2 µM final concentration), and 3.5 µL of RNAse-free MilliQ water. Primers were selected from sequences previously reported for rainbow trout and were obtained from Sigma (**Table 1**). The transcripts assayed were *tph1, tph2, 5htr_2c_, 5htr_1b_, 5htr_1a_, npy, agrp1, pomca1, cartpt, crfb*, and *actb*. The latter was used as a reference for relative quantification after it was found to show stable expression levels in tissues under the experimental conditions. Thermal cycling initiated by incubation at 95°C for 10 min using hot-start iTaq DNA polymerase activation; then, 35 qPCR steps were performed, each consisting of heating at 95°C for 15 s, 30 s at Tm-5° C and 30 s at 72°C. After the last PCR cycle, melting curves were systematically monitored (temperature gradient at 0.5°C/15s from Tm-5 to 94°C) to ensure that only one fragment from each sample was amplified. Samples lacking cDNA were run in parallel, as negative controls, to ensure primers accuracy. The efficiency of all transcripts was calculated and only values between 90% and 110% were accepted (R2 value was always higher than 0.985). The relative expression of the target genes was calculated following the Pfaffl method (41).

**Table 1:** Nucleotide sequences of the PCR primers used to evaluate mRNA abundance of transcript by RT-qPCR.

Gene	Forward primer	Reverse primer	Reference
*tph1*	AGGGAAAGATGAGAGCCTACG	GGAGAGTGCATGCTTCAG	MG015697.1 GenebanK
*tph2*	CCTTCAACACGCCTCAAAACC	ATCTTCTGGGGGAACCAAGGA	MG015698.1 Genebank
*5htr_1a_*	CACAAGGGTTTGAGAACAGGA	CATGATGATACCGAGCGTCTT	XM_021622104.1 Genebank
*5htr_1b_*	GCAATGTCAACACGGATCAC	AGGTGCGCTGAAGTGAGTCT	XM_021584469.1 Genebank
*5htr_2c_*	CCTTCGTGGCTTTCTTCATC	GAAATTTGGAGGTGGGGACT	XM_021614617.1 Genebank
*pomca1*	CTCGCTGTCAAGACCTCAACTCT	GAGTTGGGTTGGAGATGGACCTC	TC86162 Tigr
*cartpt*	ACCATGGAGAGCTCCAG	GGGCACTGCTCTCCAA	NM_001124627 Genebank
*crfb*	ACAACGACTCAACTGAAGATCTCG	AGGAAATTGAGCTTCATGTCAGG	AF296672 Genebank
*npy*	CTCGTCTGGACCTTTATATGC	GTTCATCATATCTGGACTGTG	NM_001124266 Genebank
*agrp1*	ACCAGCAGTCCTGTCTGGGTAA	AGTAGCAGATGGAGCCGAAACA	CR376289 Genebank
*actb*	GATGGGCCAGAAAGACAGCTA	TCGTCCCAGTTGGTGACGAT	AJ438158 Genebank

### 2.6 Statistical analysis

All data sets are shown as the mean ± standard error of the mean (SEM) and a significance level (α) of 0.05 was generally used. One-way ANOVA analysis was performed to assess differences among periprandial times for each parameter assayed within each cohort, ZT2 and ZT6. Post-prandial data (from 0 min to 240 min after MT) were analyzed by two-way ANOVA, which included feeding condition (fed/unfed) and time as main factors. When required, Tukey’s HSD was used for *post hoc* analysis. Statistical analyses were carried out with GraphPad Prism (version 9.0; GraphPad Software, San Diego, CA).

## 3 Results

Statistical results of the periprandial evolution of the different parameters in normally fed fish (one-way ANOVA), as well as comparisons between fed and non-fed during the postprandial time are included as supplementary material (**Supplementary Tables 1, 2**).

### 3.1 Plasma cortisol and glucose levels

**Figure 1:**
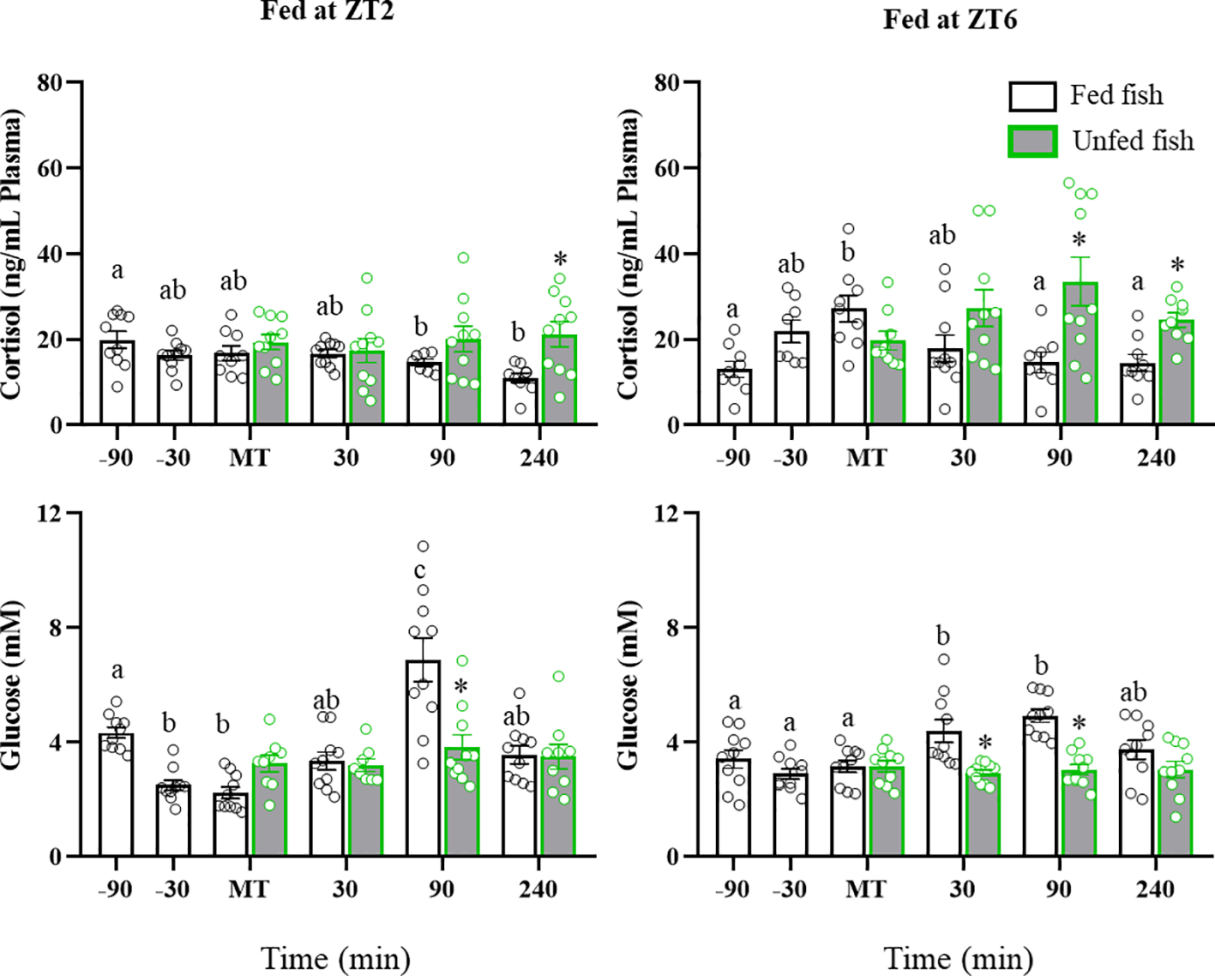
Periprandial changes and effect of fasting in plasma cortisol and glucose levels of rainbow trout normally fed at two different daily times (morning, ZT2, and afternoon, ZT6). The results are shown as the mean ± SEM of n=10 fish per group. Different letters indicate significant differences among times in the fed fish group. Asterisks indicate significant differences between non-fed and fed fish at each specific time (P<0.05).

Plasma cortisol and glucose levels are shown in **Figure 1**. Fish fed in the morning (ZT2) displayed elevated preprandial values, which gradually decreased until 240 min after MT. In contrast, cortisol levels in fish that were fed in the afternoon (ZT6; cohort 2) showed a significant increase at MT and decreased thereafter. Non-fed trout had higher cortisol levels that fed trout in both the ZT2 and ZT6 cohorts (+240 min: p<0.05 vs fed group). Plasma glucose levels in ZT2-fed trout initially decreased (-30 min before MT) and then transiently increased at +90 min. Those fed at ZT6 showed an increase in glucose levels at +30 min and +90 min, declining to basal levels at +240 min. Glucose levels in the unfed fish did not vary across the periprandial times.

### 3.2 mRNA abundance of food intake-related neuropeptides in diencephalon

**Figure 2:**
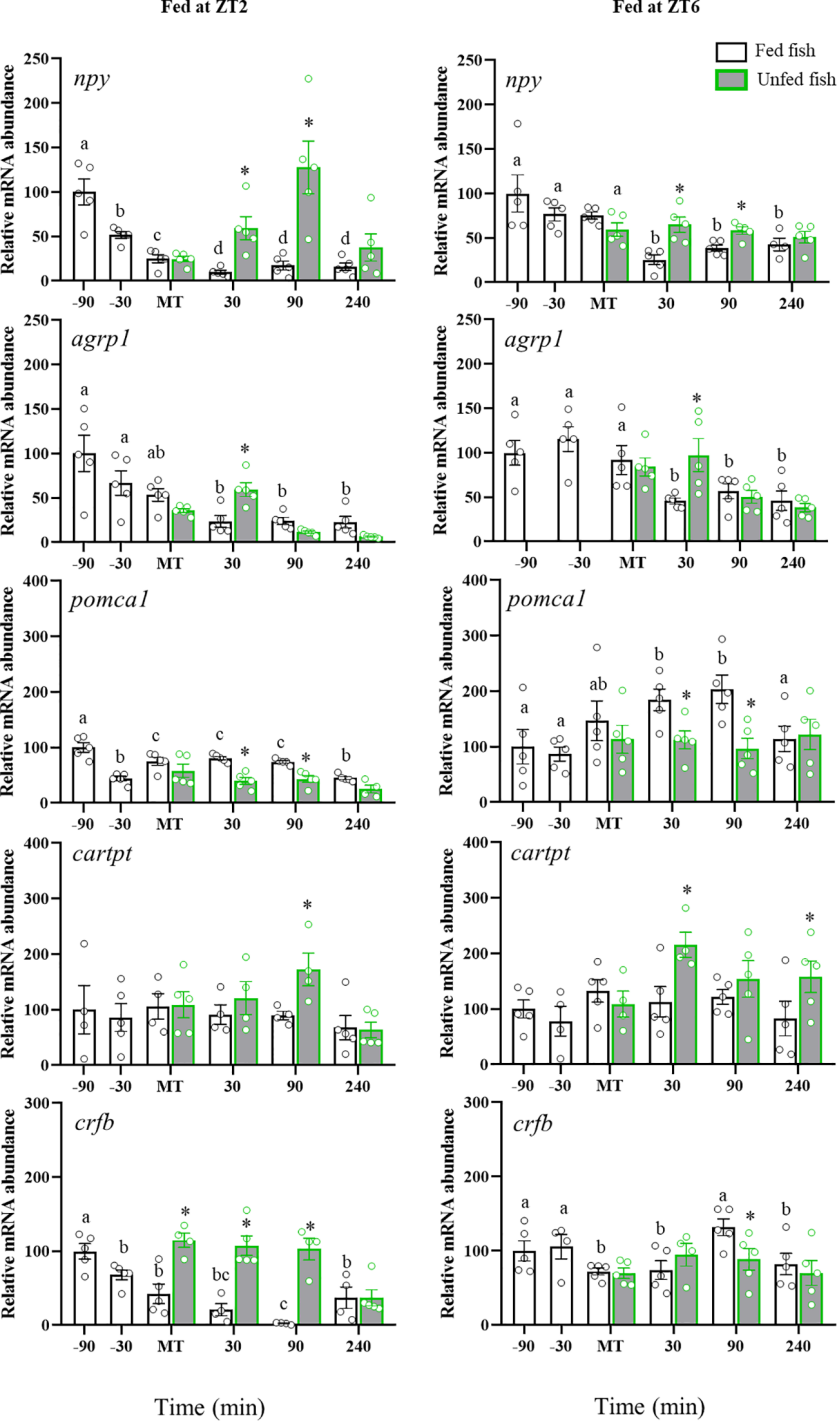
Periprandial changes and effect of fasting in mRNA abundance of regulatory neuropeptides in the diencephalon of rainbow trout normally fed at two different daily times (morning, ZT2, and afternoon, ZT6). The results are shown as the mean ± SEM of n=5 fish per group. Different letters indicate significant differences among times in the fed fish group. Asterisks indicate significant differences between non-fed and fed fish at each specific time (P<0.05).

**Figure 2** shows the mRNA abundance of *npy*, *agrp1*, *pomca1*, *cartpt* and *crfb* in trout diencephalon. The preprandial time significantly affected *npy* and *agrp1* mRNA abundance in ZT2-fed fish, showing a continuous drop from -90 min to MT. However, trout fed at ZT6 showed no preprandial changes in both peptides. After feeding, *npy* and *agrp1* levels sharply decreased in both cohorts of fish, remaining low thereafter. In the non-fed trout, this effect was not observed initially, with its levels being higher at 30 min (*npy* and *agrp1*) and 90 min (*npy*) after MT compared to the respective fed groups. After 90 min (*agrp1*) and 240 min (*npy*) from feeding, transcript levels were similar in the fed and unfed groups. As for *pomca1*, a pre-MT decrease was observed in the ZT2 group (-30 min), followed in both the ZT2 and ZT6 groups by an increase that was maintained for 90 min after MT. The non-fed trout group showed no such an increase in *pomca1*, with values remaining stable throughout sampling. No significant periprandial changes for *cartpt* were found in both ZT2 and ZT6 fed fish, but an increase in non-fed fish (ZT2: +90 min; ZT6: +30 min; p<0.05 vs. respective fed groups).

The abundance of *crfb* in diencephalon showed a decreasing trend throughout most of the sampling time in ZT2-fed fish, which was most evident after the mealtime. In fish fed at ZT6, the post-feeding decline in *crfb* content was also noted, but more attenuated. Non-fed fish showed higher *crfb* levels from usual MT to +90 min as compared to fish fed at ZT2, whereas no differences were found between non-fed and ZT6-fed fish.

### 3.3 Periprandial changes in serotonergic activity and receptors

**Figure 3:**
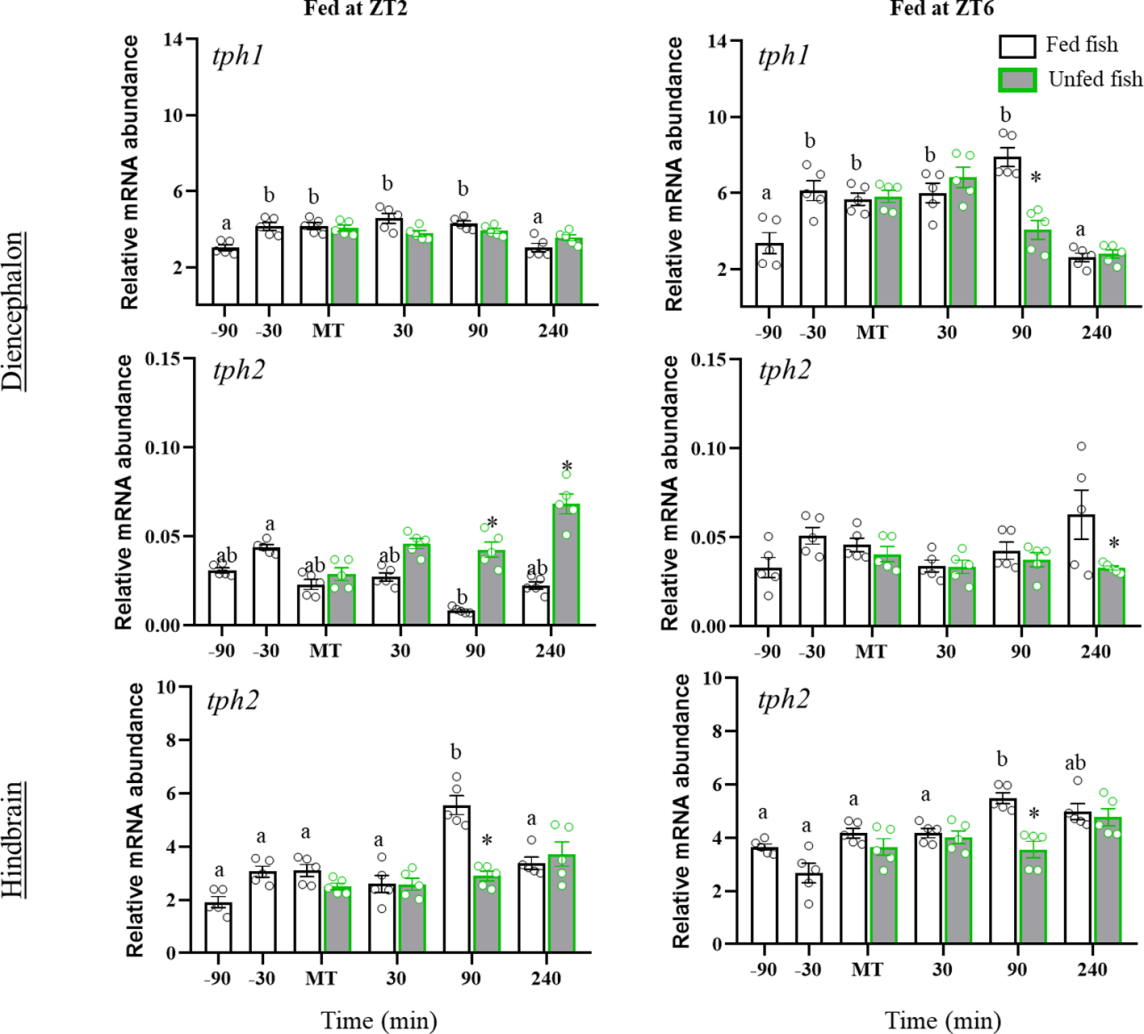
Periprandial changes in mRNA abundance of tryptophan hydroxylase 1 and 2 in diencephalon and tryptophan hydroxylase 2 in hindbrain of rainbow trout with two different feeding schedules, morning (ZT2) and afternoon (ZT6). The results are shown as the mean ± SEM of n=5 fish per group. Different letters indicate significant differences among times in the fed fish group. Asterisks indicate significant differences between non-fed and fed fish at each specific time (P<0.05).

**Figure 3** shows the mRNA abundance of *tph1* and *tph2* in the diencephalon and hindbrain of rainbow trout fed in ZT2 and ZT6, or in the respective sham-feeding groups. Fish from both cohorts showed significant time-dependent changes in diencephalic expression of *tph1* with a sustained significant increase from 30 min before MT. Non-fed fish showed differential effects as a function of daily time. Thus, *tph1* mRNA abundance was not affected in non-fed fish from the first cohort (ZT2), while those from the second cohort (ZT6) showed a significant decrease at 90 min after MT compared to the fed group (p=0.028).

Assessment of diencephalic *tph2* mRNA abundance revealed minor periprandial changes, with significantly low *tph2* mRNA levels at 90 min post-feeding in ZT2 fish, which increased in non-fed fish (from 90 to 240 min post-MT, p<0.05 vs. fed fish). Normally fed ZT6 cohort trout showed no significant periprandial fluctuations, whereas non-fed trout showed a decrease at 240 min post-MT. In the hindbrain, mean *tph2* expression was significantly higher during the postprandial than the pre-prandial period in both ZT2 (P=0.010) and ZT6 (P<0.001) fish. The *tph2* mRNA content increased transiently at 90 min post-MT in both cohorts, relative to pre-prandial and prandial values, followed by a decline to basal levels (+240 min). The postprandial increase in *tph2* mRNA levels was not observed in non-fed fish of both cohorts (ZT2 and ZT6), so it was significantly lower at +90 min than in fed fish.

**Figure 4:**
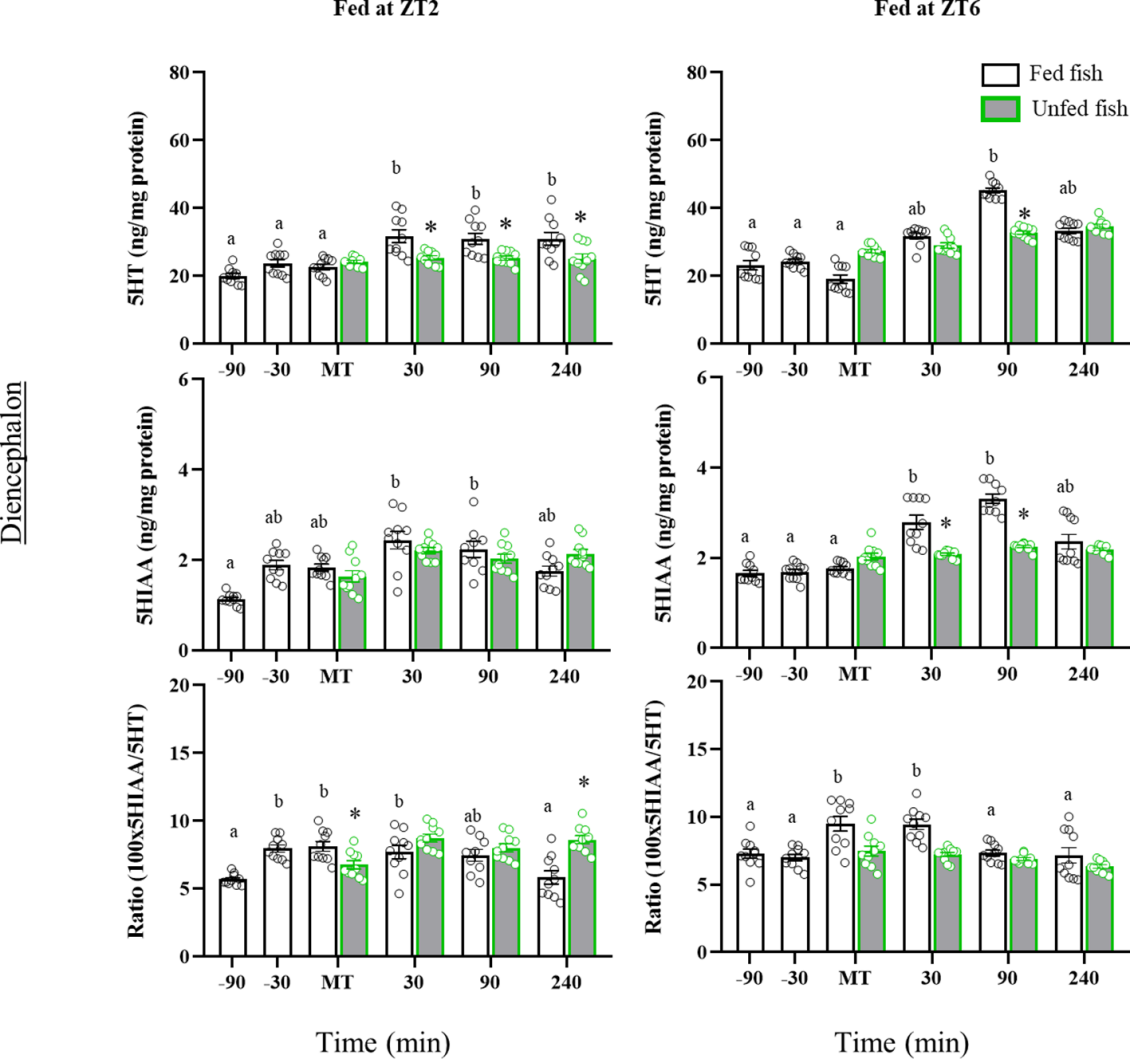
Periprandial changes and effects of fasting in the levels of serotonin (5HT) and 5-hydroxyindoleacetic acid (5HIAA), and the 5HIAA/5HT ratio in the diencephalon of rainbow trout with two different feeding schedules, morning (ZT2) and afternoon (ZT6). The results are shown as the mean ± SEM of n=10 fish per group. Different letters indicate significant differences among times in the fed fish group. Asterisk indicates significant differences between non-fed and fed fish at each specific time (P<0.05).

**Figure 4** shows the tissue contents of 5HT and 5HIAA, as well as the 5HIAA/5HT ratio in the diencephalon. 5HT content increased significantly after feeding in fish from both cohorts (ZT2: p=0.005; ZT6: p<0.001 vs pre-prandial time), whereas no postprandial changes were detected in non-fed trout. Accordingly, 5HT levels were significantly lower at 30, 90 and 240 min (ZT2) or 90 min (ZT6) after MT in non-fed versus fed trout (ZT2: p<0.001; ZT6: p=0.007). Diencephalon 5HIAA content also increased after feeding time in both cohorts, reaching significance at 30 and 90 min after MT compared to previous values. Non-fed fish in the ZT2 cohort showed the same 5HIAA content profile as fed fish, whereas in the ZT6 cohort the levels of this metabolite were less in non-fed fish than in fed fish at 30 and 90 min after MT. As for the 5HIAA/5HT ratio, increased values were found 30 min before MT (ZT2) and at MT (ZT2 and ZT6), compared to previous sampling points, remaining elevated for at least 30 min in the postprandial period. Trout in the first cohort (ZT2) that were not fed showed lower values of the 5HIAA/5HT ratio at the MT than those fed, with these levels gradually increasing over the next 240 min. A similar effect was not observed in fish from the second cohort (ZT6).

**Figure 5:**
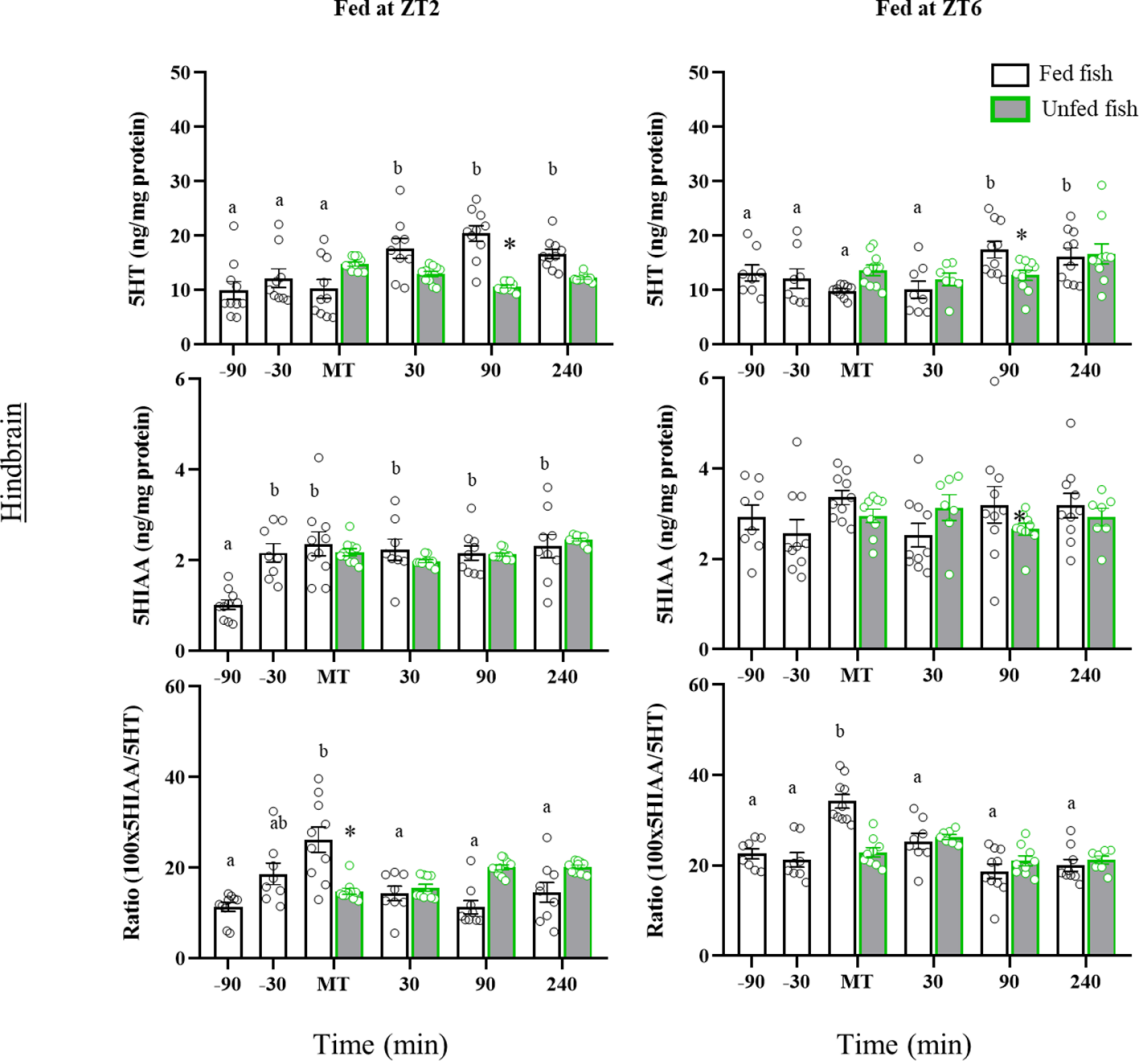
Periprandial changes and effects of fasting in the levels of serotonin (5HT) and 5-hydroxyindoleacetic acid (5HIAA), and the 5HIAA/5HT ratio in the hindbrain of rainbow trout with two different feeding schedules, morning (ZT2) and afternoon (ZT6). The results are shown as the mean ± SEM of n=10 fish per group. Different letters indicate significant differences among times in the fed fish group. Asterisk indicates significant differences between non-fed and fed fish at each specific time (P<0.05).

In the hindbrain (**Figure 5**), the 5HT content of the morning-fed fish (ZT2) increased in the postprandial period (30-, 90- and 240 min post-MT vs pre-prandial values, p=0.010). A post-prandial increase was also observed in the ZT6 group of fish (p=0.030) at 90 min and 240 min post-MT. In contrast, non-fed fish in both cohorts showed no significant periprandial changes in 5HT levels. Consequently, 5HT values were at some time lower in non-fed than in fed fish (90 min post-MT, p<0.05). The 5HIAA content in the hindbrain of morning-fed trout increased significantly before MT and remained elevated thereafter. Trout fed at ZT6 showed no significant variation throughout the entire sampling period. The 5HIAA content did not vary with time in the unfed fish of both cohorts, nor did it change relative to fed fish. The 5HIAA/5HT ratio in the hindbrain, increased transiently in MT in both cohorts, but returned to basal levels thereafter. Non-fed fish showed no such increase at MT, but 5HIAA/5HT gradually increased to significantly higher values 90 min after MT. A similar profile was not observed in fish from the second cohort (ZT6).

**Figure 6:**
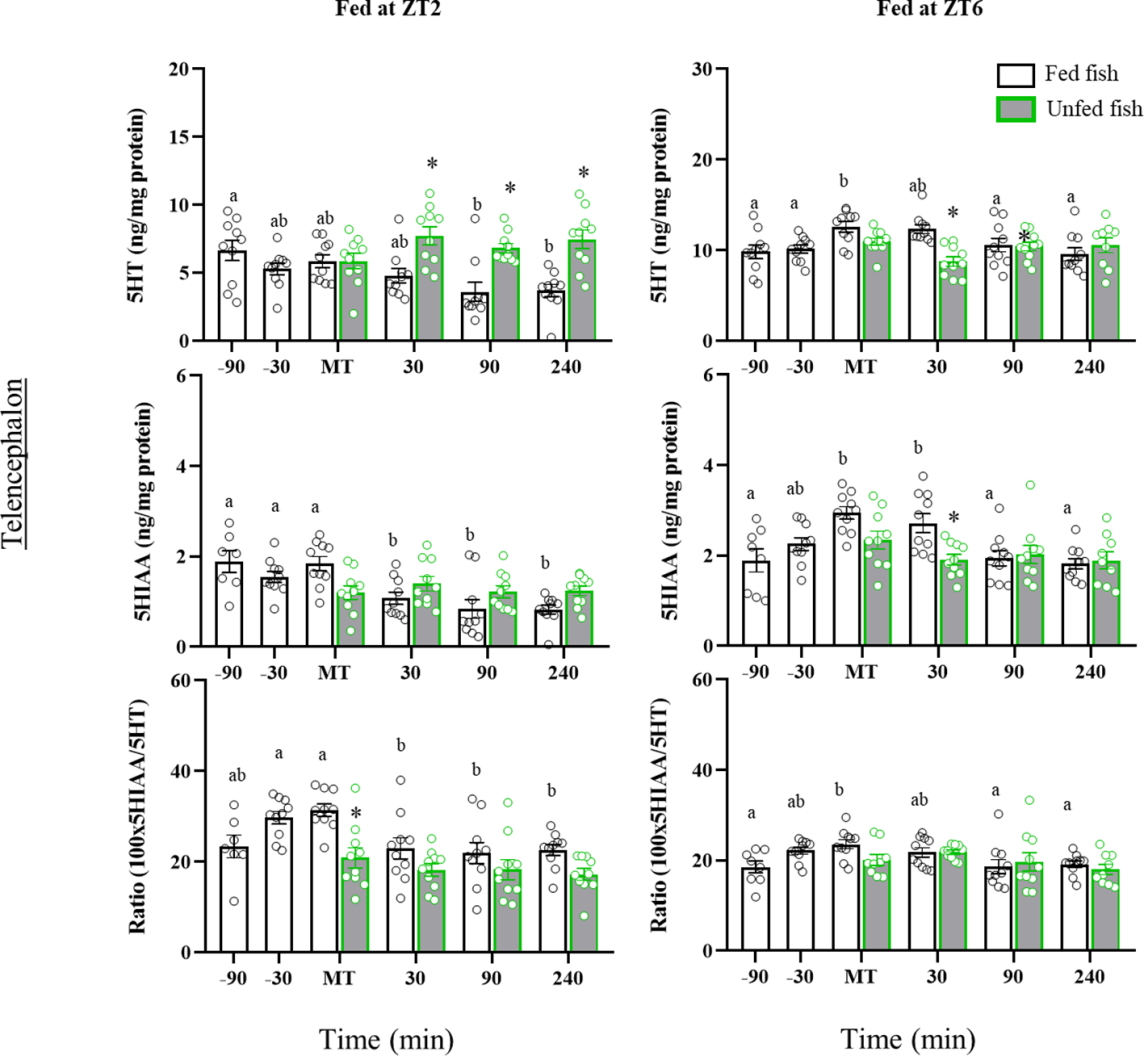
Periprandial changes and effects of fasting in the levels of serotonin (5HT) and 5-hydroxyindoleacetic acid (5HIAA), and the 5HIAA/5HT ratio in the telencephalon of rainbow trout with two different feeding schedules, morning (ZT2) and afternoon (ZT6). The results are shown as the mean ± SEM of n=10 fish per group. Different letters indicate significant differences among times in the fed fish group. Asterisk indicates significant differences between non-fed and fed fish at each specific time (P<0.05).

In the telencephalon, ZT2-fed trout showed significant postprandial decreases in 5HT (90- and 240 min post-MT) and 5HIAA (30-, 90- and 240-min post-MT) contents compared to earlier times (**Figure 6**). A decrease was also noted for 5HIAA in non-fed fish, but not for 5HT whose levels remained elevated (p<0.05 vs respective post-prandial times of fed fish). In contrast, fish fed normally in the afternoon (ZT6) had a transient prandial increase in the contents of 5HT (MT) and 5HIAA (MT and 30 min post-MT), which did not occur in non-fed fish. The periprandial profile of 5HIAA/5HT ratio values in the telencephalon was quite similar in both cohorts, with a gradual increase until the time of feeding (-30 min and MT in ZT2; MT in ZT6, p<0.05 vs. previous sampling points) and a decline to basal values after feeding. Non-fed fish lacked the increase in 5HIAA/5HT ratio shown for those fed at the time of feeding.

The mRNA abundance of 5HT receptors (*5htr1a*, *5htr2c* and *5htr1b*) was measured only at diencephalic level (**Table 2**). The expression of the genes evaluated did not change significantly throughout the periprandial period, despite a tendency of *5htr2c* to increase and *5htr1a* to decrease after MT in both cohorts. Non-fed fish showed *5htr2c* mRNA abundance profile like that of fed fish in both cohorts, although a decrease was noted at +240 min in the ZT2 cohort compared to fed fish. Likewise, *5htr1b* mRNA abundance showed a decrease at the end of the sampling period in non-fed fish (+90 min, +240 min, p<0.05 vs. fed fish) from both ZT2 and ZT6 cohorts.

**Table 2:** Periprandial changes in mRNA abundance of diencephalic serotonin receptors of rainbow trout normally fed at two different daily times (morning, ZT2, and afternoon, ZT6) or unfed.

Time (min relative to MT)		Morning (fish fed at ZT2)						Afternoon (fish fed at ZT6)					
		-90	-30	MT	+30	+90	+240	-90	-30	MT	+30	+90	+240
*5htr1a*	Fed	0.059 ± 0.010	0.042 ± 0.004	0.035 ± 0.012	0.037 ± 0.0001	0.032 ± 0.010	0.034 ± 0.007	0.045 ± 0.008	0.033 ± 0.010	0.036 ± 0.007	0.028 ± 0.008	0.040 ± 0.010	0.041 ± 0.013
	Unfed		–	0.037 ± 0.018	0.044 ± 0.011	0.051 ± 0.011	0.029 ± 0.005	–	–	0.042 ± 0.007	0.044 ± 0.009	0.043 ± 0.008	0.035 ± 0.005
*5htr2c*	Fed	0.042 ± 0.006	0.041 ± 0.013	0.041 ± 0.013	0.064 ± 0.014	0.068 ± 0.025	0.054 ± 0.015	0.046 ± 0.008	0.042 ± 0.012	0.040 ± 0.005	0.059 ± 0.013	0.062 ± 0.012	0.058 ± 0.015
	Unfed	–	–	0.026 ± 0.009	0.049 ± 0.014	0.060 ± 0.012	0.023 ± 0.005 *	–	–	0.038 ± 0.009	0.064 ± 0.009	0.059 ± 0.013	0.059 ± 0.016
*5htr1b*	Fed	0.129 ± 0.031	0.096 ± 0.012	0.081 ± 0.025	0.052 ± 0.021	0.058 ± 0.024	0.045 ± 0.007	0.112 ± 0.015	0.063 ± 0.013	0.089 ± 0.010	0.098 ± 0.030	0.126 ± 0.030	0.109 ± 0.030
	Unfed	–	–	0.062 ± 0.023	0.073 ± 0.017	0.012 ± 0.004 *	0.002 ± 0.001 *	–	–	0.116 ± 0.027	0.111 ± 0.047	0.052 ± 0.018 *	0.012 ± 0.005 *

Asterisks indicate significant differences between non-fed and fed fish at each specific time (P<0.05). The results are shown as the mean ± SEM of n=6 fish per group.

## 4 Discussion

### 4.1 Feeding influence on mRNA levels of neuropeptides in diencephalon

This study reports periprandial profiles in mRNA abundance of food intake-related neuropeptides in concert with serotonergic activity in the rainbow trout brain. The changes found in diencephalic neuropeptides are consistent with the role commonly attributed to them in the regulation of food intake in teleost fish. Thus, the highest mRNA abundance of the orexigenic neuropeptides (*npy* and *agrp1*) occurred before MT, decreasing shortly after feeding the fish, regardless of whether they were fed in the morning or in the afternoon. Meanwhile, mRNA levels of the anorexigenic neuropeptide *pomca1* increased significantly at MT and remained elevated for the next four hours. In contrast, *crfb* showed a tendency to decrease after food intake, whereas no periprandial changes were found in *cartpt*, so the involvement of these two neuropeptides in the control of routine food intake seems to be of less relevance.

The literature in salmonids points to NPY as a short-term appetite regulator, usually as a hunger signal (42–44). In rainbow trout, treatment (icv) with NPY dose-dependently increased food intake at 2 to 3 hours (45), supporting an orexigenic role. In relation to mealtime, brain expression of *npy* has been shown to fluctuate widely, with increases occurring minutes before mealtime even when different feeding frequencies are scheduled throughout the day (46). In the same line, increases in *npy* mRNA levels in the hypothalamus of trout after 3 weeks of fasting have been reported (47). Our data are consistent with the orexigenic role of NPY in rainbow trout as a higher abundance of hypothalamic *npy* mRNA was found preprandially in both morning (ZT2) and afternoon (ZT6) fed fish, with smaller differences later. Thus, *npy* transcript levels decrease sharply 90 min before MT in the morning, but 30 min after MT in the afternoon, perhaps reflecting the interference between daily fluctuations in peptide transcription and feeding-related changes. Indeed, we previously reported that *npy* mRNA levels in trout are highest during the second half of the night gradually decreasing until the onset of the light phase (37). Consequently, the high transcript levels observed in the morning, i.e., 90 min before MT (30 min after turning on the lights) could reflect that *npy* mRNA abundance was still decreasing toward basal at the time the fish were fed (2 h after turning on the lights), an effect that was not found in fish fed in the afternoon. Meanwhile, non-fed fish from both cohorts did not show a postprandial decrease in *npy* mRNA levels but even an increase (in the morning) was found compared to preprandial values. This is in line with other fish species in which *npy* mRNA abundance up-regulates in the hypothalamus of food-deprived fish (48–50). Furthermore, in fed trout the decrease in npy expression in the diencephalon after feeding is simultaneous with the increase in blood glucose levels, which was not observed in fasted fish. This is consistent with the role of NPY-containing hypothalamic neurons in sensing metabolic state (i.e., glucose levels), as previously reported for trout (51) and another teleost (52–54).

Feeding also influenced *agrp1* gene expression, with elevated levels of the transcript prior to MT and a decrease shortly after the meal. This fluctuation suggests that *agrp1* has an orexigenic role in rainbow trout, consistent with other salmonids such as Atlantic salmon (55) and coho salmon (56). Meanwhile, *agrp1* mRNA abundance in fasted trout remained transiently elevated (30 min after feeding) and decreased thereafter. This increase in fasting condition agrees with that reported in Atlantic salmon deprived of food for three days (55), although in other teleosts more days of food deprivation seem to be necessary for this increase to be observed (50, 57, 58). Changes in *agrp1* mRNA abundance were similar in the morning and afternoon cohorts, suggesting that acclimation to a particular feeding schedule does not determine the expression profile of this peptide in rainbow trout. However, as for *npy*, basal level of *agrp1* mRNA abundance was higher in the early morning than in the afternoon, consistent with peptide expression being affected by circadian fluctuations, as previously reported (37).

The melanocortin system seems to be a key player in the regulation of energy balance in fish (59). POMC neurons have been found in the hypothalamus of goldfish by *in situ* hybridization (59), while transcripts of the peptide have also been detected in the hindbrain of rainbow trout (51). Treatments with POMC-derived peptides, i.e., α-MSH or drugs acting on the MC4R clearly support that POMC plays an inhibitory role in food intake (6, 57). Changes in POMC transcript content during feeding are relatively consistent in salmonids, including the rainbow trout where *pomca1* transcript levels increased after feeding (47) and respond to increased glucose levels (51). However, fasting-related changes are more inconsistent across species and time (55, 60). In rainbow trout, differential responses in POMC transcript variants to fasting were reported, with *pomca1* being the only affected after 28-days food deprivation (61). Our data agree with the previous studies and show an increase in *pomca1* mRNA abundance at MT and postprandially (+30 and +90 min). Meanwhile, non-fed trout showed no such increase, suggesting that feeding-related signals rapidly influence hypothalamic expression of *pomca1* in rainbow trout.

Postprandial increases in *cartpt* mRNA abundance have been described in Atlantic salmon (60), goldfish (62), and catfish (63), among other teleost species, suggesting that this peptide acts as a short-term satiety signal (6). However, data in rainbow trout are scarce. This study shows that *cartpt* mRNA levels remained unchanged around the time of feeding, whereas they increased during the postprandial phase in trout that did not receive food. There are no clear reasons to explain this discrepancy, although it has been suggested that prandial changes in this peptide could be specific to the different *cartpt* genes identified in teleosts (64, 65). Even, a different role of the peptide in the short and long term has been suggested in some species (66, 67). Our present data do not support an inhibitory role of CART in food intake, but neither can it be ruled out that this peptide acts as a hunger signal under fasting conditions, which could be related to foraging (68).

CRF has been pointed as a negative regulator of food intake in teleost. In rainbow trout, studies showed that central CRF treatments stimulate the hypothalamic-pituitary-interrenal (HPI) axis to induce glucocorticoid (i.e., cortisol) secretion, which is further associated with anorectic effects (32, 34, 69). However, prandial changes in *crf* mRNA levels are scarcely known in salmonids and in general in teleost, whereas fasting decreased brain expression levels in other fish species (70, 71). In our study, *crfb* mRNA abundance enriched before the meal and down-regulated with the meal (ZT2 and ZT6), which is not clearly consistent with an anorexigenic role of this peptide. Furthermore, fasted fish did not have such a postprandial drop in *crfb*, but an increase was observed in ZT2-fed trout. Since changes in CRF are often related to fluctuations in HPI axis activity, cortisol levels in periprandial times were measured. We found an increase in cortisol levels before feed intake in trout ZT6-fed trout, which was not evident in those fed at ZT2 probably due to interference from elevated cortisol levels that in this species occur at the end of the night (72). In addition, cortisol values decreased postprandially in both fed cohorts, but not in the unfed fish. These data suggest that blood cortisol levels could be affected by feeding, increasing during mealtime in fed fish, and after mealtime in fish not fed at the expected time. This is consistent with other studies showing that the activity of HPI axis up-regulates during periods of fasting, increasing circulating cortisol levels (73, 74). Taken together, our data provide a similar picture during immediate fasting, which could be interpreted as an activation of the (eu)stress response linked to an alert before the arrival of food or to food seeking when it does not arrive at the expected time.

### 4.2 Periprandial changes of serotonergic activity in brain

In vertebrates, depolarization of serotonergic neurons is associated with increased 5HT synthesis to replenish the neurotransmitter in nerve terminals. This process involves the activation of TPH, the limiting enzyme of 5HT synthesis (12). Two TPH isoforms, TPH1 and TPH2, with different catalytic specificity and substrate affinity (75) are known in vertebrates. There is evidence that mammalian *tph1* mRNA is distributed in peripheral tissues and the pineal gland, whereas *tph2* expression is mainly restricted to the raphe nuclei of the brainstem (76, 77). However, in fish, the central serotonergic system extends to other brain structures, including hypothalamic and pretectal areas (26, 77). In rainbow trout, we have recently reported that both forms of *tph* are expressed in the central nervous system (27), albeit with important topographical differences. Whereas the major presence of *tph2* is found in the raphe and reticular formation of the hindbrain, both *tph1* and *tph2* exist in the diencephalic nuclei, with *tph1* restricted to the hypothalamic area. Our present data are consistent with the presence of both isoforms in the diencephalon, whereas *tph2* mRNA transcripts were only detected in the hindbrain. Expression levels of *tph* were negligible in the telencephalon, consistent with the absence of 5HT-producing cells in forebrain areas (27).

In this study, significant periprandial changes in 5HT content were noticed in brain regions of rainbow trout that are key in feeding regulation. In both the hindbrain and diencephalon, 5HT content increased shortly after feeding in fed fish, but not in non-fed fish, in which 5HT content was lower. In addition, a postprandial increase in 5HIAA levels in the diencephalon (30 and 90 min after feeding) was observed in both morning and afternoon fed fish, which was interpreted as an increased utilization of 5HT associated with the postprandial period. Diencephalic serotonergic activation is also supported by a higher abundance of *tph1* mRNA, the most prevalent TPH isoform in this region. In contrast, *tph2* mRNA levels decreased after the meal, although expression levels of this enzyme in the diencephalon were very low, suggesting that its contribution to serotonergic functionality is restricted. Overall, the changes in diencephalic serotonergic activity agree with previous pharmacological studies in rainbow trout in which increases in central 5HT (29, 33, 69) were associated with inhibition of food intake. Our data suggest that the inhibitory role of 5HT occurs at a time after feeding, i.e., the amine may act as a satiety signal. The fact that non-fed fish showed no alterations in periprandial 5HT levels and had a less consistent postprandial increase in 5HIAA levels than fed fish further supports the functional involvement of 5HT in postprandial satiety.

The release of monoaminergic neurotransmitters into the synaptic cleft occurs in discrete bursts and the amine is then reuptaken into the terminals and metabolized. Consequently, short-term changes in tissue contents of 5HT and 5HIAA respond to alterations in neuronal release, with the 5HIAA/5HT ratio being considered a valuable parameter to define synaptic mobilization of the neurotransmitter (40, 78, 79). However, neuronal turnover depends on the influence of several parameters, such as neurotransmitter synthesis, transport, reuptake, and degradation, which could be regulated independently. Thus, neuronal activation may result in simultaneous increases or decreases in 5HT and 5HIAA contents that do not translate into ratio alterations, so changes in these parameters should be analyzed individually. In our study, serotonergic activity was elevated at the time of food intake, as suggested by the increased values of the 5HIAA/5HT ratio, then remaining high for a long time, as showed by enhanced 5HIAA content in both the diencephalon and hindbrain. This suggests that prandial activation of serotonergic neurons is rapid and quite prolonged in time, possibly extending into the post-absorptive satiety phase.

Feeding is a complex behavioral process that also affects foraging and develops in anticipation of the next meal in the form of increased motor activity, social interactions, or the accentuation of hierarchies, among others (80, 81). The so-called meal anticipatory activity is a typical example of scheduled daily pre-feeding behavior, which in teleost is under circadian control (38, 82). Strikingly, we observed that either *tph1* mRNA abundance (ZT2 and ZT6 cohorts) or the 5HIAA/5HT ratio (ZT2 cohort) increased before the time of feeding (-30 min, compared to previous values), suggesting that diencephalic serotonergic activity already increases before the fish receives food. These results are novel relative to previous pharmacological studies and point to diencephalic 5HT being involved not only in the cessation of feeding, but also in its initiation. Our data point to a possible circadian influence driving an increase in serotonergic activity prior to feeding; however, the non-inclusion of a randomly fed group in the study precludes drawing conclusions in this regard. There is also no previous information linking the preprandial increase in hypothalamic serotonergic activity to other feeding-related behaviors. However, administration of enhancers of 5HTergic activity results in increased locomotor activity in zebrafish (83), while brain 5HT is involved in complex social processes, such as hierarchy formation, aggression, and social defeat (84), which may be exacerbated prior to feeding when animals are most active. In cavefish, it has been proposed that two neighboring hypothalamic populations of serotonergic neurons play dissimilar roles, one related to satiety and the other modulating foraging and aggression (85, 86), with these behaviors likely to be enhanced prior to feeding. Therefore, it is possible that serotonergic neurons at diencephalic locations are involved in pre-feeding behavior in teleost fish, although the specific mechanisms are unknown.

Serotonergic activity in the hindbrain also increased before the meal (ZT2-fed fish) or during the meal (ZT6-fed fish), showing increased levels of 5HIAA and/or an increased 5HIAA/5HT ratio. In addition, *tph2* mRNA abundance was significantly increased in the hindbrain during the postprandial phase. This region contains clusters of neurons homologous to mammalian raphe nuclei, which intensely express *tph2* transcripts (27). TPH2 enzyme activity is known to be more susceptible to be influenced by substrate availability than TPH1 (87), so tryptophan influx during feeding could lead to increased 5HT synthesis at sites with elevated TPH2 activity, such as mammalian raphe nuclei (76) or the hindbrain of fish (15). Serotonergic neurons in the teleost raphe region project extensively to hypothalamic areas (77), making this region susceptible to alterations in 5HT related to substrate availability. Therefore, food and the resulting increase in tryptophan availability could also affect hypothalamic 5HT content and release through its effects on 5HT biosynthesis in neuronal projections originating from the raphe. Thus, our data are consistent with a possible contribution of 5HT from raphe neurons to hypothalamic projections, which could be restricted to the postprandial time when 5HT is involved in the cessation of food intake.

The projections of dorsal and ventral 5HT neuronal populations from the hindbrain and diencephalon reach distinct forebrain groups. The changes detected in our study in serotonergic parameters in the telencephalon were relatively distinct from those found in the diencephalon. While food intake was followed (0 and 30 min) by higher levels of 5HT and 5HIAA in the afternoon-fed fish, an increase in the 5HIAA/5HT ratio was also evident at the time of feeding, but not before feeding, in the morning-fed fish. Non-fed fish did not show such an increase, so it appears that mealtime-associated signals trigger 5HTergic neuronal activation in this region. In addition, there was a decrease in 5HTergic activity after feeding in trout that ate at their feeding time, which did not appear in trout that did not receive food. These data rule out a role for telencephalic 5HT as a satiety-related signal, although it may have a role in those not receiving food at the expected time. Several 5HT receptors such as the subtypes 5HT_2A_, 5HT_1A_, 5HT_1B_ and 5HT_2C_ are expressed in fish forebrain, implicating the amine in different physiological processes (88). Our results support a role of 5HT in the forebrain distinct from that related to satiety and may include processes such as the management of sensory information related to food, locomotor activity associated with foraging or related to the reward system (89), although this deserves further investigation.

### 4.3 Linking changes in brain 5HT and feeding control

**Figure 7:**
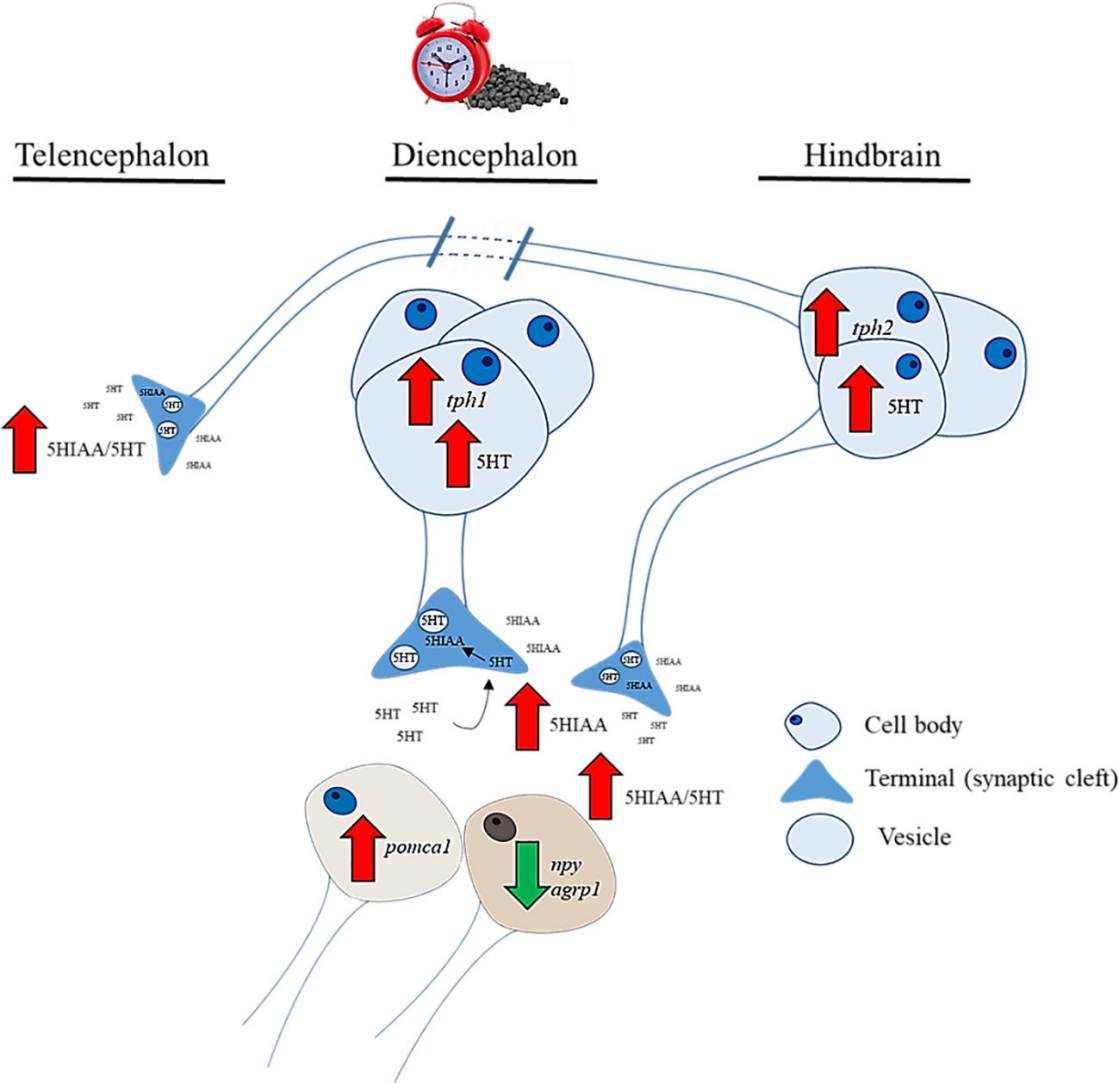
Graphical summary about the regional changes in brain serotonin functioning during feeding time in rainbow trout and proposed action on hypothalamic neuropeptides involved in food intake regulation.

Overall, the serotonergic parameters analyzed in our study indicate that feeding time is coupled with increased 5HT abundance in somatodendritic and projection areas harboring neuropeptides related to regulation of food intake (**Figure 7**). Additionally, we monitored changes in periprandial mRNA abundance of three 5HT receptor subtypes, 5HTR_1A_, 5HTR_1B_, and 5HTR_2C_, which have been shown in mammals to prominently regulate food intake (10, 11, 90). No changes in 5HT receptor mRNA abundance were found in fed trout, limiting any conclusions about a possible relationship with food intake regulation at prandial and postprandial times. In fish, mechanistic studies are not as precise as in mammals, but treatments with 5HT or drugs that stimulate 5HTergic signaling, such as fluoxetine or d-fenfluramine, reduced the rate of food intake in goldfish (28, 31) and rainbow trout (29, 30). As for the 5HT receptors mediating this action, the most consistent results point to an involvement of the 5HTR_2C_ subtype, whose agonist activation produces a decrease in food intake along with an increase in *pomc* expression in the hypothalamus of rainbow trout (33, 91). This is in agreement with mammalian data, where POMC neurons contains *5htr2c* receptors whose activation largely determines the anorectic effect of the amine. In our study, a reduction in *5htr2c* mRNA levels was found in fasted compared to fed fish, which was very late (+240 min) relative to the timing of feeding and was observed only in the morning cohort of fish. In fed fish, some tendency for a postprandial increase was found in both cohorts, but without statistical significance. Therefore, these results are still too weak to predict an involvement of these receptors in the regulation of daily feed intake in rainbow trout, which requires further studies.

Treatment with 5HTR_1A_ receptor agonists did not alter feed intake in rainbow trout (33), which is in line with the lack of periprandial changes we have found in this receptor in fed trout. There was also no change in *5htr1b* mRNA abundance in fed trout, whereas non-fed trout showed a significant down-regulation in receptor expression at 90 and 240 min after the expected feeding time, which was concomitant with a certain attenuation of serotonergic activity. In mammals, *5htr1b* is expressed in NPY/AGRP neurons of the arcuate nucleus, and its activation leads to neuronal hyperpolarization (21) and anorexigenic effects. With this in mind, it appears that delayed decrease in *5htr1b* expression in fasted trout could act as a facilitating mechanism for hunger signals once the feeding period was exceeded and there was no access to food. Although existing pharmacological evidence in trout does not allow linking a modulatory action of 5HT on npy expression in hypothalamus (30), the increase in serotonin produced by fluoxetine injections increased such expression in goldfish (31), so this possibility should not be ruled out.

In summary, our results reveal that rainbow trout acclimated to two daily feeding schedules develop a complex serotonergic response that may, in part, be influenced by circadian inputs related with the time at which the food is provided. In addition, feeding induces anticipatory activity affecting neuropeptides systems and the serotonergic system at the diencephalic level. In relation to 5HT, the results point to an inhibitory role of this neurotransmitter in food intake probably acting as a satiety-related signal, which could be mediated by interaction with hypothalamic neuropeptides. The results also suggest additional roles of 5HT during the preprandial phase in which serotonergic activity increases. Further research on 5HT neuronal networks and links with other neurotransmitter systems is necessary to expand our knowledge of the involvement of the brain 5HT in feeding behavior in fish.

## Data availability statement

The datasets presented in this study can be found in online repositories. The names of the repository/repositories and accession number(s) can be found in the article/**Supplementary Material**.

## Ethics statement

The animal study was approved by Animal Ethics Committee of the University of Vigo. The study was conducted in accordance with the local legislation and institutional requirements.

## Author contributions

MC and JM conceived and designed the experiments. MC, RC, and ML-P carried out the experiments and analyzed the samples. MC, JM, MA, JC-R, and JLS interpreted the data and drafted the manuscript. Funding acquisition and project administration: JLS and JM. All authors contributed to manuscript revision, read, and approved the submitted version.
