# Supplementary material for: Unraveling the periprandial changes in brain serotonergic activity and its correlation with food intake-related neuropeptides in rainbow trout Oncorhynchus mykiss
Source: Front Endocrinol (Lausanne). 2023 Aug 24;14:1241019. doi: 10.3389/fendo.2023.1241019 (PMC10491422; doi:10.3389/fendo.2023.1241019)
Supplement: Supplementary file 1 [file DataSheet_1.pdf]

Supplementary Material

Mauro Chivite, Rosa M. Ceinos, José M. Cerdá-Reverter, Jose L. Soengas, Manuel Aldegunde3, Marcos A. López-Patiño, Jesús M. Míguez*

*** Correspondence to J.M. Míguez jmmiguez@uvigo.gal**

DATA AVAILABILITY

The datasets presented in this study can be found online in the following repository link: https://datadryad.org/stash/share/V8sd1DUjrfMQSn4zQefoM793tR6Vz-zSIgiIN37avOE

Supplementary Figure S1: Diagram of rainbow trout brain with the target tissues dissected in this study.

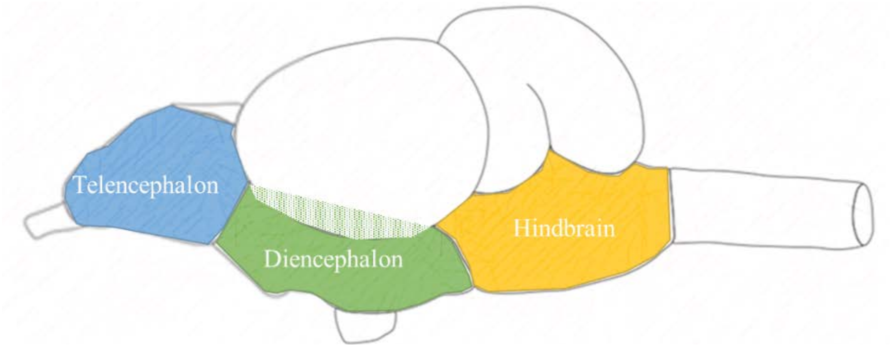

Supplementary Table S1: p-values of the one-way analysis of variance of the parameters analyzed at periprandial time within cohorts of trout fed at ZT2 and ZT6.

	**Hormones and metabolites**		**Neuropeptides mRNA**					**Serotonergic system**														
								Enzymes mRNA			Content and activity									Receptors		
	Plasma		Diencephalon					Diencephalon		Hindbrain	Diencephalon			Hindbrain			Forebrain			Diencephalon		
	**Cortisol**	**Glucose**	***npy***	***agrp1***	***pomca1***	***cartpt***	***crfb***	***tph1***	***tph2***	***tph2***	**5HT**	**5HIAA**	**Ratio**	**5HT**	**5HIAA**	**Ratio**	**5HT**	**5HIAA**	**Ratio**	***5ht1a***	***5ht1b***	***5ht2c***
**ZT2**	0,007	<0,001	<0,001	0,005	0,003	-	<0,001	0,021	0,033	0,010	0,005	0,004	<0,001	0,010	0,021	0,025	0,002	0,001	0,021	-	-	-
**ZT6**	0,022	<0,001	0,015	0,019	0,023	-	0,036	0,005	-	<0,001	<0,001	0,001	0,020	0,030	0,042	0,003	<0,001	0,026	0,041	-	-	-

Supplementary Table 2: p-values of the principal components (Feeding, Time) of the two-way analysis of variance for parameters assessed during postprandial time (from MT to +240 min) in cohorts of rainbow trout fed at ZT2 or at ZT6.

				ZT2			ZT6		
				**F**	**T**	**F/T**	**F**	**T**	**F/T**
**Hormones and metabolites**		plasma	Cortisol	0,011	-	-	0,036	-	0,043
			Glucose	0,006	<0,001	<0,001	0,003	0,015	0,009
**Neuropeptides mRNA**		diencephalon	*npy*	<0,001	0,003	0,002	0,035	0,023	0,012
			agrp1	-	<0,001	0,001	-	0,018	-
			pomca1	<0,001	<0,001	-	0,008	-	-
			cartpt	-	0,036	-	0,017	-	-
			crfb	<0,001	0,033	0,004	-	0,026	-
**Serotonergic system**	Enzymes mRNA	diencephalon	*tph1*	-	<0,001	0,010	0,028	<0,001	<0,001
			tph2	<0,001	<0,001	<0,001	0,001	0,039	0,005
		hindbrain	*tph2*	0,002	<0,001	<0,001	<0,001	0,004	0,007
	Content and activity	diencephalon	5HT	<0,001	<0,001	<0,001	0,007	<0,001	<0,001
			5HIAA	-	<0,001	0,013	<0,001	<0,001	<0,001
			Ratio	0,043	0,015	<0,001	<0,001	<0,001	-
		hindbrain	5HT	<0,001	0,004	<0,001	-	<0,001	<0,001
			5HIAA	-	-	-	-	-	-
			Ratio	<0,001	<0,001	<0,001	0,017	<0,001	<0,001
		forebrain	5HT	<0,001	0,001	<0,001	0,002	0,001	<0,001
			5HIAA	0,004	0,001	<0,001	<0,001	0,001	<0,001
			Ratio	<0,001	<0,001	<0,001	-	<0,001	-
	Receptors	diencephalon	5ht1a	-	-	-	-	-	-
			5ht1b	0,034	-	-	0,026	-	-
			5ht2c	-	-	-	-	-	-
